# Preictal neuronal and vascular activity precedes the onset of childhood absence seizure: direct current potential shifts and their correlation with hemodynamic activity

**DOI:** 10.1117/1.NPh.10.2.025005

**Published:** 2023-04-25

**Authors:** Mina Nourhashemi, Mahdi Mahmoudzadeh, Claire Heberle, Fabrice Wallois

**Affiliations:** a Université de Picardie Jules Verne, Inserm U1105, GRAMFC, CURS, Amiens, France; b Amiens University Hospital, Pediatric Neurophysiology Unit, Amiens, France

**Keywords:** general spike and wave discharges, absence epilepsy, seizure, direct current potential, electroencephalography, functional near-infrared spectroscopy, diffuse correlation spectroscopy, cerebral hemodynamics, cerebral blood flow

## Abstract

**Significance, Aims:**

The neurovascular mechanisms underlying the initiation of absence seizures and their dynamics are still not well understood. The objective of this study was to better noninvasively characterize the dynamics of the neuronal and vascular network at the transition from the interictal state to the ictal state of absence seizures and back to the interictal state using a combined electroencephalography (EEG), functional near-infrared spectroscopy (fNIRS), and diffuse correlation spectroscopy (DCS) approach. The second objective was to develop hypotheses about the neuronal and vascular mechanisms that propel the networks to the 3-Hz spikes and wave discharges (SWDs) observed during absence seizures.

**Approaches:**

We evaluated the simultaneous changes in electrical (neuronal) and optical dynamics [hemodynamic, with changes in (Hb) and cerebral blood flow] of 8 pediatric patients experiencing 25 typical childhood absence seizures during the transition from the interictal state to the absence seizure by simultaneously performing EEG, fNIRS, and DCS.

**Results:**

Starting from ∼20  s before the onset of the SWD, we observed a transient direct current potential shift that correlated with alterations in functional fNIRS and DCS measurements of the cerebral hemodynamics detecting the preictal changes.

**Discussion:**

Our noninvasive multimodal approach highlights the dynamic interactions between the neuronal and vascular compartments that take place in the neuronal network near the time of the onset of absence seizures in a very specific cerebral hemodynamic environment. These noninvasive approaches contribute to a better understanding of the electrical hemodynamic environment prior to seizure onset. Whether this may ultimately be relevant for diagnostic and therapeutic approaches requires further evaluation.

## 1 Introduction

Neurovascular coupling (NVC) is essential to supply the energy demands of brain tissue during both normal and abnormal events, such as epileptic discharges.^1^^–^^5^ Childhood absence epilepsy is a type of idiopathic nonconvulsive generalized epilepsy characterized by episodes of 3 to 4 Hz generalized spike and wave discharges (GSWDs),^6^ causing a temporary and involuntary arrest of consciousness and unresponsive staring for a few seconds.^7^ Despite a number of studies performed in animal models and humans over the last several years, the key questions concerning how seizures initiate and propagate are yet unresolved. It is still unclear how the neuronal system communicates to spontaneously achieve a synchronous signal change in all channels^8^^–^^11^ notably the hemodynamic changes that surround the episodes of absence epilepsy, which are still debated.^12^^–^^14^ Understanding the dynamics of neuronal and vascular networks that lead to the transition to seizures and identifying reliable signatures for this process is an area of active research.^15^ An approach that may be promising to study the initiation of seizures is to study neuronal and vascular changes and the direct current (DC) shift immediately before the onset of a seizure, as hemodynamic changes related to neuronal activation, and DC shifts might concern the activation of not only the synchronized pyramidal cells but also nonsynchronized interneurons and glial cells, which are not monitored by electroencephalography (EEG) recordings. Based on neurovascular coupling, which is the key interface that links the neuronal and the vascular systems, functional optical neuroimaging techniques can distinguish cortical hemodynamic [oxyhemoglobin (HbO), deoxyhemoglobin (HbR), cerebral blood flow (CBF)] and neuronal processes involved in the adaptation of a physiological function to a pathological condition, such as in absence.^16^^–^^19^

### 1.1 Cortical DC Shift

Previous studies revealed that the onset of ictal DC shifts in intractable focal epilepsy precede that of ictal electrophysiological signals, notably ictal high-frequency oscillations, suggesting that the occurrence of ictal DC Shifts may indicate a role of glia in seizure generation.^20^ DC potentials are used to monitor the entire frequency range of extracellular field potentials,^21^ which are comprised of sustained shifts and slow fluctuations, as well as faster waves superimposed on deviations from the baseline.^21^ It has been shown that negative DC shifts associated with seizure activity originate from a mixed generator of neurons and functionally related glial cells.^22^ The current generator for negative EEG waves of the α and γ range, as well as sharp waves, is more superficially located, including the apical dendritic membrane.^23^ But the direction of the shifts of surface potentials shows no simple relationship to unit activity, as seen during spike-wave complexes by EEG. Most sharp, surface-negative potentials of the EEG were found to coincide with excitatory unit activity, whereas the “slow wave” correlated with a pause in neuronal activity.^16^^,^^21^^,^^22^^,^^24^ No simple correlation between surface potentials and cellular activities has been demonstrated and neurons, as well as functionally related glial cells, from different layers of the cortex are reflected differently in surface potentials.

### 1.2 Cerebral Hemodynamics

Hemodynamic imaging using functional near-infrared spectroscopy (fNIRS) and diffuse correlation spectroscopy (DCS) is a powerful noninvasive approach that can measure changes in hemodynamic activities throughout the brain. CBF increases or decreases during absence epilepsy has been discussed in many studies.^25^ Either no clear effect on CBF was demonstrated,^26^ CBF varied among patients,^27^ or a CBF decrease after the spike-wave onset,^28^^–^^30^ followed by an increase in CBF during the postictal phase.^30^ Complex change in cerebral hemodynamic was observed in the frontal area, with an increase in HbO starting few seconds before the onset of spikes and waves, followed by deoxygenation.^4^ Frontal ictal hyperperfusion and interictal hypoperfusion have also been shown in absence seizures using 99mTc-hexamethylpropylenamine oxime brain single photon emission computed tomography (SPECT).^31^

The good temporal resolution of simultaneous recordings of EEG, fNIRS, and DCS provides a feasible global approach to trace the dynamics of cerebral blood oxygenation and flow signal changes associated with preictal, ictal, and interictal epileptiform activity. fNIRS takes advantage of a living tissue’s absorption properties in the near-infrared range to measure changes in the local concentrations of HbO and HbR through the intact skull^32^^,^^33^ in response to neuronal activation known as neurovascular coupling.^19^^,^^34^^,^^35^ The relative changes in CBF measured by a DCS device are proportional to the relative changes in tissue blood flow related to the motion of red blood cells.^2^^,^^3^

We hypothesized that the emergence of absence epileptic seizures results from a disturbance of the functional balance between various neuronal, vascular, and metabolic compartments. Hence, a multimodal approach, using fNIRS and DCS combined with DC-EEG, was applied to better understand (1) the neuronal and vascular mechanisms and dynamics of the various compartments prior to the 3-Hz GSWD, the biomarker of absence seizure; (2) whether such multimodal analysis can provide new information on the mechanisms that propel neurons to the hyperactivation and hypersynchronization that result in an absence epileptic seizure; and (3) the interactions between the previously described hemodynamic changes in HbO and HbR prior to the onset of absence seizures and the changes in CBF and EEG-DC potential.

## 2 Materials and Methods

This study examined 8 children, ranging in age from 6 to 10 years (6 males and 2 females) presenting, in total, 25 GSWDs on EEG. According to MRI and/or CT, all had normal brain anatomy. This study is part of the French Public Hospital Clinical Research Project. The study was approved by the Amiens University Hospital local ethics committee according to the guidelines of the Declaration of Helsinki of 1975 (CPP Nord‐Ouest2 No. IDCRB: A01513-40). Parents were informed about the study and provided their written informed consent.

### 2.1 Multimodal Data Acquisition (Simultaneous HD-EEG, fNIRS, and DCS Measurements)

**Fig. 1:**
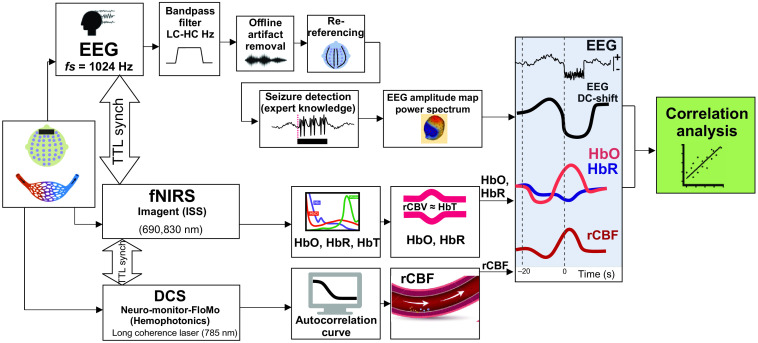
Diagram of the measured hemodynamic activity (HbO and HbR by NIRS, rCBF by DCS) and schematic presentation of the pipeline applied to the EEG signal, correlation between hemodynamic responses, and EEG DC-shift.

Data were recorded by a combined multimodal approach using EEG, fNIRS, and DCS (Fig. 1). An external transistor–transistor logic (TTL) trigger was used between the electrophysiological (EEG) and hemodynamic measurement systems (fNIRS, DCS) to synchronize the recording devices [Fig. 1(b)]. All recordings were performed in a dark room. The optical devices are time-multiplexed (i.e., sources can be turned on and off to allow brain tissue to be illuminated for either DCS or fNIRS at any given time). All major events observed by DCS were also recorded by the EEG and fNIRS devices [Fig. 1(b)].

### 2.2 Electrophysiological Measurement

#### 2.2.1 Neural activity measurements by high-density EEG

**Fig. 2:**
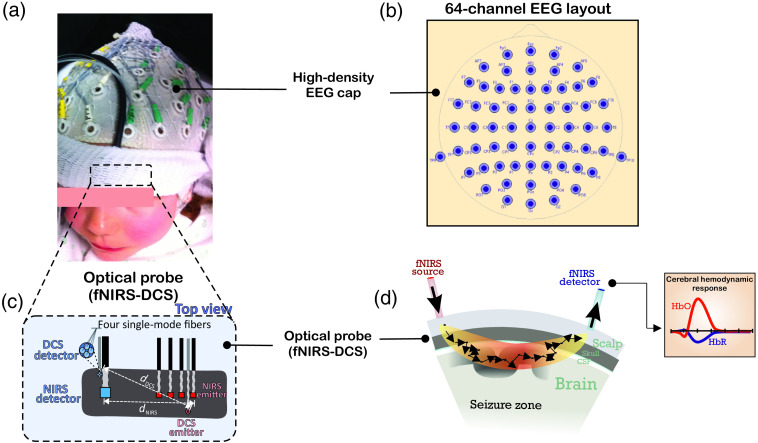
(a) High-density EEG cap and optical probe (NIRS-DCS). (b) Schematic representation of the location of the EEG and optical probes on a child’s head. (c) View of the combined optical (fNIRS DCS) probe constructed with a flexible hypoallergenic material and composed of two sectors. For the DCS probe, the tips of the source and detector fibers were tightly held in place at a 90 deg angle by a flexible rubber material. For the fNIRS probe, the four emitters (red squares) were arranged on a multidistance patch away from the detector (Blue square), creating four measurement points (channels) over the frontal area. (b) 64-channel EEG layout and (d) schematic diagram of the photons propagation. NIR-light is generated and guided to the head by optical fibers or cables. Another fiber bundle or cable directs diffusively reflected light from the head to the detectors. A light detector captures the light resulting from the interaction with the chromophores (e.g., HbO, Hb), following a crescent-shaped path back to the surface of the skin.

High-density EEG was acquired using 64 electrodes to measure electrical potentials resulting from underlying neuronal activity [Fig. 2(a)]. The EEG was recorded using Ag/AgCl surface electrodes and a Cz reference at a sampling rate of 1024 Hz, amplified by A.N.T.® (Enschede, The Netherlands). A high-pass filter of 70 Hz and a notch filter at 50 Hz were applied. The electrode impedances were maintained below 5  kΩ. Video EEG recordings were performed during quiet arousal. Patients were monitored by video to detect artifact movements to exclude any altered data. Off-line analysis was performed using BESA research (ver. 7) and in-house MATLAB scripts for all signals. The exact timing of the onset of the electrical seizure was determined from the EEG-filtered data (0.5 to 30 Hz) by an experienced, board-certified electroencephalographer (F.W.). The artifacts from the EEG data were visually detected and rejected. The mean duration between the onset and end of the absence seizure (i.e., SWDs) was 12±4  s.

#### 2.2.2 Power spectra and time-frequency analysis

The frequency power spectra were applied to the seizure-marked region to quantify the frequency content of the seizure. The seizure epoch was delineated by markers and the fast Fourier transform (FFT) was then computed for this segment. The previously calculated FFT spectrum can be redisplayed. Topographical maps of the power spectrum were calculated using BESA research. Maps of the power spectrum results were based on average referenced data. This was necessary to ensure that the electrodes were given equal weights in the nonlinear transformation to power. The selected seizure segments were preprocessed for the FFT in two steps: (1) a window function was multiplied by the data to attenuate the amplitudes at the ends to zero and (2) the number of samples was increased by interpolation (if necessary) to convert it to a power of 2. The seizure block constituted 80% of a window of 100% width. A cosine squared (cos²) window of 20% of the total width was applied at the edges to attenuate the signal amplitude to 50% at the edges of the marked block and zero at the edges of the window. Then, we took the number of time samples in the window and selected the next higher power of 2. Using spline interpolation, the data in the window were converted to this larger number of points and the FFT applied.

Time-frequency representation (TFR) was performed for frequencies between 1 and 8 Hz to characterize the changes more precisely in neuronal activity occurring during the seizure. TFR was performed according to the procedures described by Hoechstetter et al.^36^ and implemented in BESA Research®. TFRs were first computed for each seizure epoch by applying complex demodulation.^37^ The TFR was calculated over each seizure epoch. Frequencies were sampled (Gaussian filter) in 0.25-Hz steps, and latencies were sampled in 200-ms steps, corresponding to a time-frequency resolution of ±0.354  Hz and ±315  ms for each time-frequency bin (full width at half maximum).

### 2.3 Optical Measurements (fNIRS and DCS)

The optical measurements for monitoring changes in light absorption (changes in [HbO] and [HbR]) were performed by fNIRS (Imagent^®^, ISS Inc.). Near-infrared (NIR) intensity fluctuations were measured by DCS (Neuro-Monitor-FloMo, Hemophotonics S.L., Spain).

#### 2.3.1 Cortical oxygenation measurements by frequency-domain spectroscopy (FD-NIRS)

A multichannel frequency-domain-based optical imaging system (Imagent, ISS Inc., Champaign, Illinois, United States) was used to measure [HbO] and [HbR] concentration changes. We used 8 of the 32 intensity-modulated laser diodes at two wavelengths (λ=690  nm and 830 nm) coupled to optical fibers and 1 of the 4 gain-modulated photomultiplier tube (PMT) detectors to collect the signal separately at both wavelengths. The modulation frequency of laser intensity was 110 MHz and the cross-correlation frequency for heterodyne detection was 5 kHz. Reflected light collected by the PMT was then demodulated. Its mean intensity (DC), modulation amplitude (AC), and phase were determined. The average output power of the lasers was ∼0.5  Mw, and the optical acquisition rate was 9.7656 Hz (about one sample every 100 ms). HbO and HbR are chromophores that absorb light at different wavelengths. The modified Beer–Lambert law was applied to the two wavelengths (λ=690  nm and 830 nm) to convert signal intensities into relative changes in (de) oxyhemoglobin concentration.

We did not use the complete frequency-domain fNIRS (FD-fNIRS) device capabilities. Unfortunately, calibration of the instrument is necessary when using the FD-fNIRS approach with multiple light sources to calculate the absolute hemoglobin concentration, notably when the device is used in different protocols and setups, which necessitate unscrewing the emitters and detectors fibers, thus requiring a calibration at bedside.The optical coupling efficiencies between the lasers and fiber-optics, as well as between the fiber-optics and sample, are not equal because the light emitted by the various laser diodes are neither of identical intensity nor entirely in phase. Therefore, before doing actual measurements, the probe (and the instrument) should be calibrated by measuring a phantom with known optical characteristics. The probe can be moved to an unknown sample (or tissue) after calibration so that “calibration corrections” can be applied while taking the measurement.

In practice, since the coupling efficiency of the fiber-optic connections can fluctuate, calibration is required each time a probe is connected to the device. In addition to the burden of performing the measurements using three synchronized modalities (EEG-fNIRS-DCS), accurate calibration of the fNIRS by nonexpert medical staff in front of patients is very challenging. This is not only inconvenient and very challenging in a clinical environment but also two (perhaps incorrect) presumptions are made: (1) that the relative intensities and phases of the sources will remain constant and (2) that the optical coupling efficiencies will not notably vary because movement artefacts are rare in absence epilepsy. In practice, thermally induced changes in laser diode intensity and phase can cause significant signal drift (low frequency noise).

For these reasons, we used an FD-NIRS device to measure relative values rather than the absolute hemoglobin concentration.

#### 2.3.2 Intermodality interference between EEG and optical measurements

From a fiber-less to fiber-based probe: before starting the study and consistent with optical probe design, we investigated the effects of crosstalk between modalities and concurrent measurements of EEG-fNIRS using a commercial device [NIRO-200NX (Hamamatsu Photonics, Japan)]. This instrument uses three light-emitting diodes, with wavelengths of 735, 810, and 850 nm, and two detection photodiodes to measure light attenuation at different distances from the source.

The results of the first fiber-less hybrid measurement showed nonnegligible induced crosstalk from the fNIRS into the EEG signals. Thus, a new hybrid probe approach was considered to decrease the effects of intermodality interference between fNIRS and EEG. After a stage of development within the study, we introduced a new probe design based on optic fibers.

A number of new features, including optic fiber design, were incorporated in the final probe scheme. As the light source is remote, the fiber transmits the light and isolates the direct contact of the active component from the light source to the scalp. Optical fibers are useful as they are nonconductive and electrically isolate the patient being measured and eliminate intermodality crosstalk into the EEG channels introduced from switching source currents.

#### 2.3.3 Intermodality interference between optical measurements (fNIRS, DCS)

That emitter crosstalk can occur when two or more light sources are simultaneously used to actively illuminate the photodetector. To eliminate such crosstalk in our study, time division multiplexing techniques were used by enabling a single emitter during a single time slot. The optical devices were time-multiplexed (i.e., sources can be turned on and off to allow brain tissue to be illuminated for either DCS or NIRS at any given time). All major events observed by DCS were also recorded by the EEG and NIRS devices. An external event trigger synchronized the acquisition devices. That the various measuring systems must be accurately synchronized to simultaneously measure hemodynamic activity. Synchronization was achieved by an external event trigger that produces TTL signals via a parallel port (line point terminal) using an in-house MATLAB^®^ code. This system simultaneously produces markers on both systems to indicate the beginning and end of data acquisition. Synchronization was performed using the level triggering mode. In this mode, the device monitors the A/D converter for an increase in the voltage level applied to the marker/trigger pins. When the appropriate increase in voltage is reached (TTL “high” signal), the measuring system records the event.

#### 2.3.4 Cortical blood flow measurements by DCS

A DCS device was used to noninvasively quantify changes in CBF. The DCS measures blood flow by optical modality using intensity fluctuations of NIR light.^38^ The light scattered by the movement of red blood cells inside the vessels causes temporal fluctuations of the detected light intensity. The time lag of such fluctuations is quantified by the intensity-time autocorrelation function of the detected light.^39^ The correlation diffusion equation is applied to fit the autocorrelation function to calculate a CBF index.^40^ The neuro-monitor-FloMo (Hemophotonics SL, Spain) consists of a narrow-band continuous-wave (CW) laser (785 nm, Crystalaser Inc., Nevada, United States) with a long coherence length (>50  m), with fast photon-counting avalanche photodiodes (SPCM-AQR-14-FC, Pacer Components Inc., United Kingdom) and a channel autocorrelator board (Flex03OEM-4CH, Correlator Inc., New Jersey, United States). In our setup, one emitter–detector pair was positioned at the left hemisphere cortex (at the same holes as for the fNIRS) and fixed at 90 deg. The system uses CW lasers in the NIR range (∼785  nm), with an acquisition rate of 0.3921 Hz (one sample every 2.55 s). The light was delivered to the brain surface via multimode fibers and detected by four single-mode fibers away from the source ($d_{\mathrm{DCS}}=2\;\mathrm{cm}$).

#### 2.3.5 Hybrid fNIRS-DCS probe

The optical fNIRS-DCS probe used for this study was specifically designed to overcome the challenges of simultaneous fNIRS-DCS recording. Recording sites on the patient’s head and a diagram of the probes are shown in Fig. 2(a). We recorded the children in a quiet dark room in a reclined position. The probe was smoothly secured to the front of the patient’s head with straps and foam padding. Because of the high density cap and the size of the patch, the fNIRS-DCS patch was placed as close as possible to the frontal border of the EEG cap. The probe was based on a 3-mm-thick rectangular rubber plate attached to optodes. The probe consists of 90 deg bent fibers (DCS) and prism-coupled (fNIRS) fibers that were placed into flexible, light-sealed pads attached to the head by straps. The DCS part of the probe is composed of a series of fiberoptic (400  μm core diameter) sources (light emitting) and one fiberoptic (light collecting) detector bundle (3-mm diameter) placed at the end of the rows of fiberoptic emitters, creating four measuring points (channels) over the frontal area. The FD-NIRS part of the probe is composed of four emitter fibers (with multidistance in one row) connected to 690- and 830-nm lasers [Fig. 2(a)]. The emitting tips were “clad” in plastic tubing and introduced into holes in a rubber block. The input tip of the detector bundle was firmly attached in contact with the prisms. The probe was held in place by a headband to minimize any light interference. Special care was taken to ensure that the light from the source of one modality did not saturate the detectors of the other.

#### 2.3.6 Optical data analysis (cerebral hemodynamic activity)

The concentrations of HbO and HbR were calculated using the modified Beer–Lambert law,^41^ based on the difference in absorption between two wavelengths (690 and 830 nm), using the MATLAB-based Homer2 toolbox (https://homer-fnirs.org/). Hemoglobin (HbT) concentrations were calculated by summing the HbO and HbR concentrations. A z-score-based algorithm was used to reject artifacts from optical signals. As individual features, such as skull thickness, influence signal strength, the signal was first homogenized for each participant by computing a z-score across all measurement periods for each channel. A z-score>4 in any channel was considered to be an artifact, in which case the entire artifact time-window was excised from the data for all channels. Segments during which the data exceeded a z-score of 4 were discarded (prespecified threshold as outliers; in μ±4σ, the expected fraction of data inside the range is 0.99). Thus, above this threshold, the data are reflected by larger amplitudes than the brain hemodynamic data: the lower the threshold, the more stringent the artifact detection, and the higher the threshold, the less stringent. This threshold appears to be satisfying, as it works on almost all optical signals.^35^ The HbO and HbR concentrations were band-pass filtered (0.01 to 0.3 Hz, order 4, zero-phase-lag Butterworth filter) to reject very slow drift of the baseline and cardiac artifacts.

The mean value of the regional CBF (rCBF) signals recorded from four paired source-detectors was calculated. The remaining cleaned hemodynamic signals were band-pass filtered (0.03 to 0.5 Hz) using a zero-phase filter (Butterworth, order 3) to eliminate high frequency physiological noise (>0.3  Hz, e.g., slow drifts, arterial pulse oscillations, respiratory rate) and, for DCS, very low frequency (<0.03  Hz) related to instruments noise.

Hemodynamic changes around the time of seizure activity (time-locked to the EEG) were studied by averaging the hemoglobin concentration data within a 90-s window (−30 to 60 s from the onset of the seizure). The changes in HbO and HbR that occurred around the time of the seizure were compared to the baseline segment (lasting −30 to −20  s before T0). Finally, a grand average of hemodynamic responses HbO, HbR, and rCBF was calculated for all subjects. To compare the hemodynamic responses to the baseline (−30 to −20  s), statistical analyses (Student’s t-test) were performed on the amplitude of HbO, HbR, and rCBF to highlight the effect of the seizures on the hemodynamic signals.

#### 2.3.7 Correlation analysis

We used robust Spearman correlations to test the relationships between the hemodynamic variable (i.e., HbO) and DC-EEG amplitude. The temporal correlation between the fNIRS HbO signal and DC-EEG was calculated using Spearman correlation coefficients across all seizures. The correlation coefficients were converted to z scores using Fisher’s transform to assess the temporal correlations at the group level.

## 3 Results

### 3.1 Electrical Brain DC Shift

**Fig. 3:**
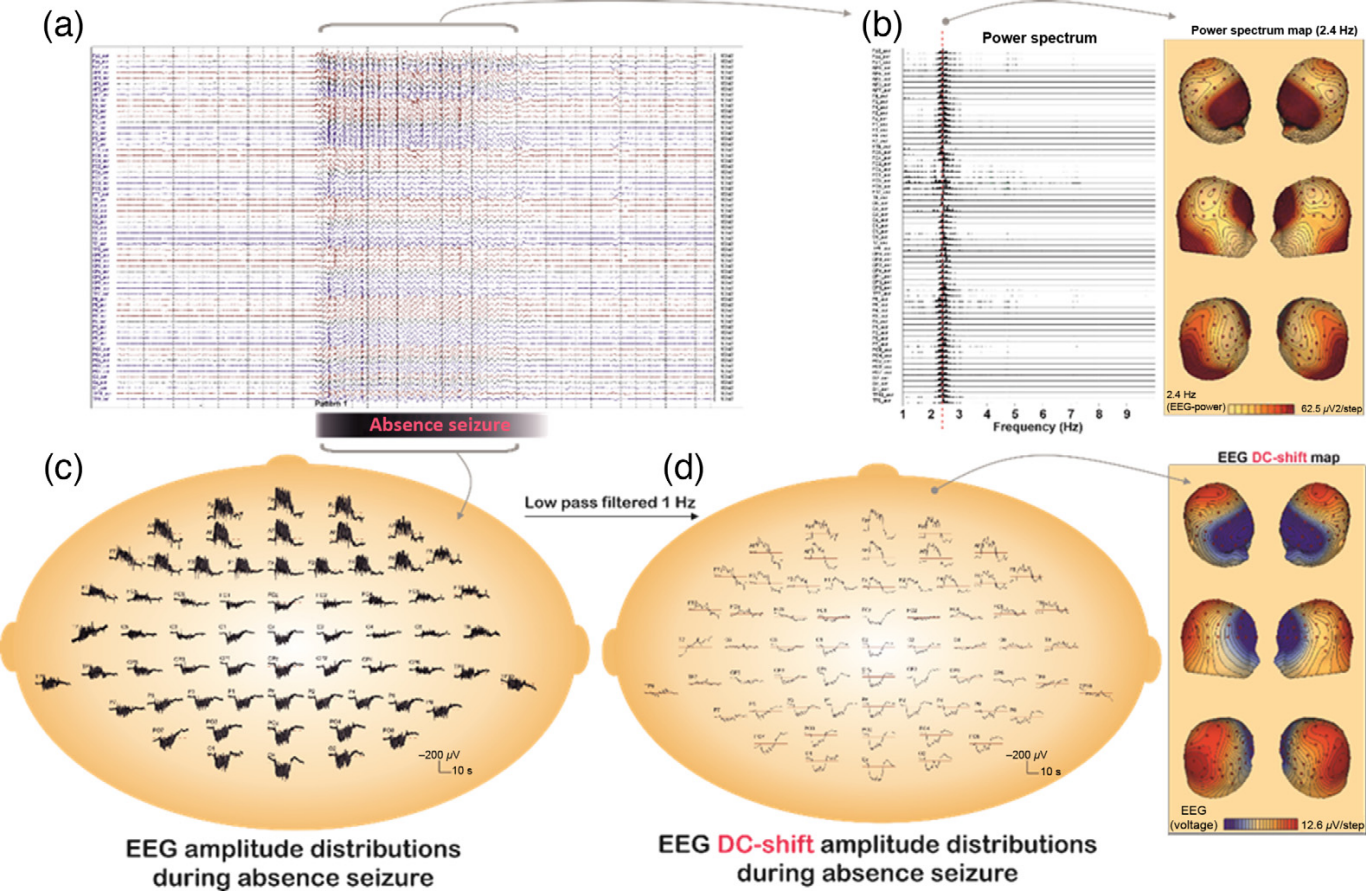
(a) Sample of recorded SWDs. (b) Power spectra and related EEG topographic maps at 2.4 Hz for the seizure segment shown in Fig. 3(a). GSWDs are most prominent over the frontal areas. Power increases greatly over 2 to 3 Hz, with the maximum power at 2.4 Hz. (c) EEG amplitude distributions during an absence seizure. (d) EEG DC-shift amplitude distributions during an absence seizure and related EEG topographic maps.

All SWDs recorded were typically found to be accompanied by an ictal DC shift, with frontal positivity and posterior negativity. The DC shift reached its maximum positivity over the midline in the frontal area and declined abruptly toward the more central regions, where it gradually inverted toward the more posterior electrodes, where it became negative. This DC shift lasted for the duration of the GSWD before returning to baseline and is depicted in Fig. 3.

### 3.2 Cerebral Hemodynamic Pattern

**Fig. 4:**
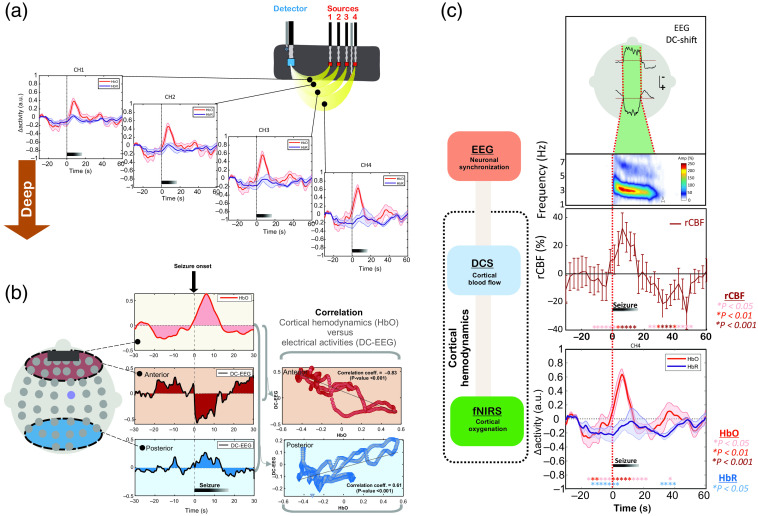
(a) A preictal decrease in cortical hemodynamics precedes seizure onset. With respect to seizure onset, there is a preictal decrease in HbO and rCBF, which then increases after seizure onset. The grand average of the regional hemodynamic responses [changes in HbO and HbR measured by multidistance NIRS (ISS)] of the onset of GSWDs at 0 s is presented. The time duration shown covers from 30 s before to 60 s after the onset of the GSWDs; the first 10 s was defined as the baseline. The lines indicate the onset and endpoint of the GSWDs. Note that the amplitudes of the hemodynamic changes decrease when the distance between emitter and detector decrease. (b) Grand average correlation between changes in the EEG DC-shift and HbO signal for 25 GSWDs. The HbO and EEG DC-shift show a negative correlation in the frontal region (correlation coff. = −0.83, P<0.001) and a positive correlation in the posterior regions (correlation coff. = 0.61, P<0.001). (c) EEG signal power around 3 Hz, showing the changes from the beginning to the end of the seizures. Average time-frequency dynamics of SWDs are shown for EEG channel (Hz). The grand averages of the rCBF (DCS) and HbO/HbR (fNIRS) associated with the seizure are shown.

We analyzed the hemodynamic activity of 8 absence epileptic children for a total of 25 seizure events (Fig. 4).

The pre- and postseizure changes in oxy- and deoxyhemoglobin concentrations showed a similar pattern for all children, in agreement with our previous results.^4^ During GSWDs, the hemodynamic changes associated with seizures were simultaneously recorded by EEG and fNIRS (Fig. 3). Hemodynamic changes started ∼20  s before onset of the seizure and consisted of a significant (P<0.05) decrease in HbO, HbR, and rCBF 15 s before the onset of the EEG burst (Fig. 4). During GSWDs, a significant increase in HbO, starting before the onset of the GSWD, with a peak at 8 s after the onset of the GSWD and a return to baseline at 16 s, accompanied by a nonsignificant change in HbR, revealed a pattern of high oxygenation during the seizures (P<0.001) (Fig. 4). rCBF started to increase at the onset (T0) of the seizure (P<0.001), with a peak at 8 s, and then returned to baseline by the end of the seizure (Fig. 4). A significant long-lasting decrease in CBF was observed after the GSWD simultaneously with small significant changes in HbO and HbR. The dynamic changes in HbO–HbR for different distances between the emitters and detector are presented in Fig. 4. The most prominent hemodynamic changes were observed for the longer distance, suggesting that they are more pronounced in deeper layers and therefore reflect the cortical origin of hemodynamic changes. The hemodynamic response supports pollution (or crosstalk) from deeper structures that progressively disappear from the deep to superficial layers. Only the changes related to the longer distance were therefore analyzed (Fig. 4).

## 4 Discussion

A multimodal approach that combines EEG, fNIRS, and DCS was developed to analyze the dynamics of the various neuronal and hemodynamic compartments that contribute to the emergence of absence seizures. In the present study, we focused on simultaneous changes in neural and vascular activities that occur near the onset of epileptic absence seizures, especially those preceding the GSWD by a few seconds. The first alteration in the measured signals was observed in the hemodynamic compartments (HbO, HbR, CBF) nearly 20 s before the onset of the absence seizure,^42^ simultaneous with the occurrence of a DC shift

### 4.1 Hemodynamic Changes during the Pre-seizure Period

Using SPECT, Baumgartner et al.^43^ showed that rCBF increases in the epileptic temporal lobe several minutes before observable changes in the EEG or clinical onset of the seizure. They suggested that rCBF changes observed on peri-ictal SPECT scans cannot be considered a mere consequence of the electrical seizure activity but may rather reflect a change in neuronal activity that precipitates the initiation of the ictal state. Using combined EEG and fMRI, DeSalvo et al.^44^ found focal BOLD increases in specific areas of the somatosensory cortex and thalamus several seconds before seizure onset in the preictal period in anesthetized Wistar rats. However, due to relatively low temporal resolution, such techniques are not suitable for rigorous temporal investigation of preseizure dynamics, which often require continuous longtime monitoring of the period prior to, during, and after seizures, with relatively high sampling frequency.^45^ Nonetheless, these results confirm that changes in hemodynamic activity might start before seizure onset.^46^

Using fNIRS, we identified complex changes in the hemodynamic parameters that started to become significant ∼20  s before the onset of the seizure (Phase I), in agreement with previous studies performed by fNIRS on humans.^4^ Zhao et al.^47^ showed that local changes in intrinsic optical signals precede seizure onset by ∼20  s. They showed a simultaneous decrease in hemoglobin oxygenation and a decrease in cerebral blood volume (CBV) within the gyrus of known ictal onset. In addition, they showed preictal vessel constriction as early as 5 s prior to seizure onset in an animal model using a two-photon microscope.^48^

In certain cases, visual inspection may reveal changes in the amplitude and frequency of EEG background activity several seconds before the onset of SWDs.^49^^,^^50^ This is often concomitant with discreet changes in the level of vigilance that occurs before SWDs. In partial epilepsy, many methods have been suggested for detecting the preictal state in EEG recordings by linear and nonlinear time series analysis.^51^^–^^59^ In absence epilepsy, a few studies have described changes in synchronization between the electrical activities of different cortical regions before the onset of absence seizures.^50^^,^^60^^,^^61^ Such early changes in both synchronization of the neuronal network^50^ and hemodynamics occur within the same time interval before the GSWD. Here, we went a step further by analyzing the various compartments simultaneously.

### 4.2 Phase I: Hypoxic Stress for the Surrounding Neuronal Network

With no *a priori* assumptions about the direction of the relationship between the dynamics of the neuronal and vascular systems and using a baseline distant from seizure onset, the very initial changes started 20 s before seizure onset (being significant at −15  sec), and were characterized by a concomitant decrease in HbO and HbR, corresponding to an initial decrease in blood volume and CBF. These results are compatible with hypoxic stress in the surrounding neuronal network that lasts for 20 s, ending just before onset of the seizure, which likely participates in the set of events that contributes to changes in the dynamics of the surrounding neuronal activity. This may correspond to the early decrease in hemoglobin oxygenation, called the “epileptic dip,” when using intrinsic optical signals.^62^^–^^64^ Thus, phase I, corresponding to a decrease in oxygenation, with a decrease in HbR (P<0.05) before the GSWD, supports diffuse interictal deactivation. The effect of the filtering and averaging methods we used on the observed early decrease in hemodynamic parameters was addressed in our previous studies related to interictal spikes.^65^^,^^66^ The averaging and deconvolution methods yielded a similar delay in the hemodynamic changes. Our modeling and experimental results demonstrated that low pass filtering with cut-off frequencies above 0.3 Hz had little effect on the hemodynamic response function (HRF) profiles estimated by the two techniques.^67^ In addition, these early hemodynamic changes occurred simultaneously with changes in neuronal network synchronization.^50^ It is therefore improbable that these early changes are related to the filtration and averaging method used in the present paper and in the previous one by Roche et al.^68^

### 4.3 Phase II: Simultaneous Increases in CBF and HbO_2_

Starting from the trough of CBF and HbO (∼2  s before seizure onset), CBF increased concomitantly with an increase in HbO. This corresponds to the classical positive BOLD signal in response to neuronal activity.^69^ In this classical model of NVC in response to neuronal recruitment, such as during a seizure, a switch in the interaction between the neuronal and vascular systems occurs, during which the vascular dynamics (HbO and CBF) become linearly and nonlinearly modulated by neuronal activity.^70^

During phase II, the simultaneous increase in oxygenation and CBF during the seizures peaked before the end of the GSWD. The increase in oxygenation and rCBF during the GSWD support a vasodilatation effect related to the NVC in absence seizures. The increase in rCBF, increase in HbO, and decrease in HbR indicate that the oxygen reactivity was adequate to meet the increase in metabolic demand during the seizures.

### 4.4 Phase III: BOLD Signal

The hemodynamic signals (HbO, HbR, and CBF) are modulated for 50 s. Given the dynamics of the neurovascular coupling, with a peak of HbO and CBF occurring 7 s after neuronal activation of the seizure, a second significant increase in HbO and decrease in CBF occurred at approximately 30 s. Autonomous oscillation of the vasculature dynamics (elasticity), with a vascular undershoot, should not be completely ruled out, even if the shape of the HbO and HbR curves (out of phase) argue for coupling with neuronal activity. Such modulation of HbO and CBF may explain certain apparently contradictory results (positive versus negative BOLD signals) when using a method with poor temporal resolution (on the order of 1 s), such as fMRI.^71^^–^^73^

Our results are partially consistent with our previously described responses to GSWDs.^4^ In both studies, a preictal phase is described, with an onset of the increase in HbO starting before onset of the GSWD. During the ictal phase, an increase in HbO is described in both studies. However, this increase in HbO during GSWDs was only observed in the previous paper in the three patients that showed long-duration GSWDs and was not observed for short duration GSWDs. The main differences consisted of the decrease in HbO that was consistently observed in all patients after seizure offset, which was not retrieved in the present study. Consistent with a study performed using PET^74^ but in contrast to those performed using laser Doppler flowmetry,^29^ CBF increased and peaked 7 s after the onset of the GSWD.

In the previous study, a significant decrease in CBF was observed within 20 to 50 s after seizure offset. This was not associated with markedly significant changes in HbO or HbR in the present study. In accordance with our previous study,^4^ a similar tendency was observed, i.e., a small increase in HbO and a small significant decrease in HbR, suggesting that the neuronal network presents secondary activation that is not associated with an increase in CBF 0 to 40 s after the GSWD.

In contrast to EEG, fNIRS signals are not restricted to the impact of activation of neurons oriented in particular directions (i.e., pyramidal cells). Glial cells do not provide changes in potentials that can be recorded by EEG. Interneurons, which are highly solicited in the functioning brain, have a closed field structure and are therefore electrically and magnetically invisible, although they are known to be highly metabolically active and might require oxygen.

Changes in brain activity around GSWDs can entrain cerebrovascular dynamics, and subsequently, perturb ongoing dynamic fluctuations in local rCBF and oxygenation. GSWDs are abnormal responses of cortical neurons to thalamocortical loops, with different periods of increased cortical excitation that are followed by different periods of cortical inhibition. Thus, a first scenario is that rCBF may decrease when the inhibition of neuronal activity exceeds that of excitation, a second scenario is that rCBF may increase due to the additional neuronal activity (i.e., higher duration of excitation than inhibition), and a third scenario is that rCBF shows no change in cases of balanced inhibition and excitation of neuronal activity. Interestingly, in a previous study, we showed changes in synchronization (hyper- or desynchronization-specific for one child) occurring a few seconds before the GSWD.

### 4.5 Electrical Brain DC Shifts

Changes in DC potential, comprising fast fluctuations and slow shifts, represent objective signs of neuronal processes in the brain, what are thought to be a “generator” of slow potentials of the brain. As an EEG abnormality does not strongly suggest a focal epileptogenic area, ictal DC shifts (positive or negative) can delineate the epileptogenic area. Slow DC potential shifts might be useful in recognizing focal onset in patients with clinically generalized seizures, the spike and waves that are bilateral from their onset, generalized, usually most prominent over the frontal areas, and occurring at a rate of 3 to 4 Hz (Fig. 3).

Our findings confirm the occurrence of slow shifts in potentials accompanying the spike and wave complexes in the EEG. We observed constant DC shifts of the SWD, contrary to what has been reported in other studies.^75^^,^^76^ The lack of a change in the DC shift may have been related to measurements by an AC amplifier, incomplete exploration of the head surface in single or dual-channel recordings, or due to voltage amplitude cancellation between equipotential areas in bipolar derivations.

It is difficult to determine the origin of the DC shift, whether it is (1) the result of neural events measured from large neuronal populations, (2) related to a glial, vascular, humoral, or metabolic origin, or (3) the result of a composite of several such factors. However, it is accepted that the neural origin of at least some DC shifts can be related to electrical events for which the neural origin has long been established. Surface DC shifts recorded externally during spike and wave activity might be generated by summated current flow toward the cortical surface. Theoretically, such an event could be generated by either preferential hyperpolarization in the deep cortical layers or preferential depolarization in superficial cortical structures.^77^

There are several possible explanations concerning the generator structures involved for this difference. However, most findings suggest a significant contribution of glia potentials to seizure-related DC shifts in the outer cortical zones. This interpretation is substantiated by measurements of the extracellular potassium concentration in various cortical layers, as well as by direct recordings of glia depolarization during and after an epileptic event.^22^^,^^78^^,^^79^ Overall, experimental observations suggest that the negative DC shifts associated with seizure activity originate from a mixed generator of neurons and functionally related glial cells. The contribution of each of these elements to the compound response may vary in different brain regions depending on factors, such as the relative density of the generator structures and the actual increase in the local K+ concentration.

### 4.6 Cortical DC Shifts Associated with Changes in Gas Pressure, pCo_2_, pH, and/or Glial Potentials

Changes of gas pressure in blood and tissue have been found to exert a considerable influence, not only on EEG waves in the conventional frequency band but also on the slow components of the DC potential. This applies to changes in ${\mathrm{pCO}}_{2}$ as well as ${\mathrm{pO}}_{2}$.^21^^,^^22^ An increase in the ${\mathrm{pCO}}_{2}$ in blood and tissue is generally accompanied by significant deviations from the DC baseline. Both positive and negative shifts have been reported to occur in various animal species.^21^^,^^22^ A decrease in the ${\mathrm{pO}}_{2}$ in cortical tissues usually evokes a negative shift of the DC baseline.^22^^,^^24^

Furthermore, the fraction of glial potentials in a complex neuronal/glial response can be enhanced if hypercapnia is combined with hypoxia. Thus the polarity of the DC shifts elicited by repeated elevations of ${\mathrm{pCO}}_{2}$ sometimes change from positive to negative, coincident with a reversal in the initial increase in tissue ${\mathrm{pO}}_{2}$.^21^ In this case, the negative DC deviation may result, in part, from a decrease in neuronal membrane potential, but a considerable portion appears to be attributable to glial depolarization due to a greater increase in extracellular $K^{+}$ activity. It is possible that such mechanisms account for some of the opposing observations concerning the polarity of DC responses to hypercapnia and acidosis. Aside from neurons and glial cells, a number of other structures, such as the blood–brain barrier, should be considered as generators of DC displacement caused by changes in ${\mathrm{pCO}}_{2}$ and pH.^80^^,^^81^

An initial decrease in HbO and CBF has also been described in other studies.^70^^,^^82^^,^^83^ Whether such hemodynamic activity is truly preictal or the expression of subtle underlying nonpyramidal neuronal or glial activity is also unclear and will require more sensitive measurements of both signals. Thus, preictal changes may not be evoked by neurons but rather astrocyte- or pericyte-medicated signaling or local potassium and neurotransmitter/neuropeptide release.^84^^–^^88^

We thus propose two hypotheses: (1) the neurogenic hypothesis: changes in the neuronal dynamics were not visible because they involved non-neuronal activity or that of astrocytes, which do not develop an electrical signature; (2) the hemo-neural hypothesis: hemodynamics may affect neural activity through direct and indirect mechanisms (i.e., hemodynamics alter the gain of local cortical circuits).^89^ Consistent with this hypothesis, Moore and Cao^89^ suggested that functional hyperemia, the “overflow” of blood to a brain region during neural activity, provides a spatially and temporally correlated source of regulation, modulating the excitability of the local neuronal circuit.^89^

Alternatively, slight changes in spontaneous vascular (and/or hemodynamic) oscillations in an atmosphere of interictal hypermetabolism in an epileptic site, as observed in focal penicillin-induced epilepsy in rats,^90^ may be the initial cause, with initial relative hypoxic stress before the GSWDs that trigger complex synaptic and nonsynaptic events. This in turn may induce neurovascular coupling, which might modulate the hemodynamic oscillations.

Epileptic seizures are generally considered to be a sudden alteration of neuronal function that occurs randomly and unpredictably. This view should change, as several studies,^4^^,^^47^^,^^48^ including the present study, indicate that seizures can be preceded by detectable changes in the neuronal and vascular dynamics of the brain. Seizures can be preceded by detectable hemodynamic changes that may reflect changes in a shift of the dynamics and excitability of epileptic networks toward the ictal state. Most of the observations described above were obtained in a simplified brain region that comprises only limited parts of the epileptic network. Using whole head EEG-fNIRS-DCS systems, further studies could better investigate the mechanisms that propel neurons to seizure onset much more extensively, involving other regions and making it possible to address interactions between regions.

### 4.7 Limitations

Performing multimodal analysis requires the use of various imaging modalities that use different measuring scales and have different temporo-spatial resolution. This may introduce a bias in the analysis of the results when comparing the different modalities. Due to their complexity, it is not always possible to perform simultaneous recordings, notably for functional optical imaging, for which crosstalk between different devices prevents simultaneous measurements. Performing multimodal monitoring is also challenging, because it can be difficult to maintain the stability of each recording over a long period, notably because of the difficulty in keeping the child immobile. We paid particular attention to probe similar brain structures and placed the EEG cap and optical probes as similarly as possible on the head of each subject for the recordings of the various modalities to minimize probe localization errors, but it was impossible to place them in exactly the same position. Our main objective in this study was to characterize neuronal and vascular interactions between the various neuronal and vascular compartments. Further studies are required to investigate specificity according to different seizure patterns and to the type of epilepsy in different species. Changes in systemic physiological parameters, such as changes in blood pressure, respiration, blood flow, and oxygenation in extracerebral tissue layers, can also affect the fNIRS signal and may confound cerebral hemodynamics. In the current study, the preictal changes in both HbO and HbR were unlikely to originate solely from extracerebral systemic activity. However, we cannot completely exclude the influence of these confounding factors. Hence, one limitation of the current study was not incorporating short distance measurements. Heating and sweeting related to it could lead to changes in coupling, which may be misinterpreted as changes in HbO and HbR. In addition, using the slope of the signal of a multidistance optical recording rather than the amplitude of the signal, the results would have not been impacted by heating. But such artifact changes related to heating are unlikely to be also related to the onset of the absence as heating occurs rather continuously during the recording. Thus, to control for this potential confounder, it would likely be worthwhile to incorporate a short-distance channel in the fNIRS configuration for future fNIRS measurements to take the full advantage of the frequency domain capabilities using a multidistance approach to evaluate changes in the absolute values of HbO and HbR, following calibration on a phantom.

Despite the use of several multidimensional methods, each which has its own limitations, the biological processes behind the GSWD were not completely monitored and require further studies, notably at deeper levels of the brain.

Functional optical measurements (fNIRS, DCS) are limited to the outer cortex and have only moderate spatial resolution (around 1 cm).^17^^,^^19^^,^^35^ The measurement depth in fNIRS and DCS mainly depends on the source-detector distance.^2^^,^^3^^,^^39^ The depth of the cortical surface relative to the scalp differs from one region of the cortex to another but does not exceed 2 or 3 cm. Unlike fMRI, functional optical measurements cannot provide information about anatomical landmarks and are not suitable for examining structures, such as the thalamus or for studying relationships between cortical and subcortical structures. It is important to note that the relationship between the cerebral hemodynamic signal recorded by these functional optical measurements and the associated neuronal activity is complex. First, cortical hemodynamic changes (i.e., the fNIRS or DCS signal) may reflect modifications in firing rates and subthreshold activity and so may not distinguish between areas of neuronal inhibition and excitation.^91^^–^^93^ Second, cortical hemodynamic changes reflect the activity of many neurons and astrocytes and are thus unable to differentiate between large changes of activity in a small number of cells and small changes in a large number of cells.^91^^–^^93^ Furthermore, although microrecordings of the local field potential and multiunit activity provide a direct measure of neuronal activity, fNIRS and DCS measurements of (de)activation merely provide an indirect indication of neuronal activity based on local cerebral hemodynamics and metabolic function.^2^ In functional optical measurements, activation is caused by higher/lower local blood flow and/or volumes (rCBF and regional CBV) containing higher/lower oxygen concentrations.^2^ Therefore, neither fNIRS-DCS nor the other cerebral hemodynamic/metabolic imaging techniques can determine which neuronal processes occur at the seizure site; any activation or deactivation measured by functional optical measurements cannot be readily equated to neuronal excitation or inhibition. Despite these issues, fNIRS and DCS can, nevertheless, provide an overall evaluation of cortical activity.

## 5 Conclusion

In the present study, we highlighted the dynamical changes occurring around the absence seizure. We notably demonstrated that early changes in HbO and HbR preceed the onset of GSWDs suggesting that GSWDs can be initiated by various not only neuronal but also vascular and metabolic processes that perturb the epileptic network. Multimodal approaches provide further insights into the complex temporal interactive mechanisms between neuronal and vascular compartments that propel the brain network to an absence seizure. Thus, it may be of interest to consider the preictal phenomena and the neuronal, vascular, and metabolic factors that drive the system dynamics toward the critical point of transition to the alternative state of the seizure.

In the present study, we observed a negative correlation between the DC shift and the hemodynamic changes suggesting that neurons, glial cells, and the blood–brain barrier, which form functional units, may act as a mixed generator, considering that the slow components of cortical DC potentials reflect changes in the level of excitation and excitability of the cortex. Despite such a variety of elements, neurons can be considered as an essential generator of the DC shifts that occur under physiological and pathophysiological conditions.

## Code, Data, and Materials Availability

All code, data, and materials that support the findings of this study are available on request from the corresponding author. The data are not publicly available due to privacy and ethics restrictions.
